# The role of minimally invasive surgery in paediatric mediastinal masses and thoracic tumours

**DOI:** 10.3332/ecancer.2025.2027

**Published:** 2025-11-13

**Authors:** Jaime Shalkow-Klincovstein, Cristian Puerta, Andrew M Davidoff

**Affiliations:** 1 ABC Cancer Center, Sur 136 No 116, Mexico City 01120, Mexico; 2 Department of Surgery, University of California, San Diego, 9300 Campus Point Drive, La Jolla, CA, 92093, USA; 3 Division of Surgery, St. Jude Children´s Research Hospital, 262 Danny Thomas Place, Memphis, TN 38105, USA; a https://orcid.org/0000-0003-0056-3455; b https://orcid.org/0000-0002-0953-0150; c https://orcid.org/0000-0001-7900-2794

**Keywords:** paediatric minimally invasive surgery, thoracic tumours, mediastinal masses, paediatric oncology, paediatric surgical oncology

## Abstract

The Role of Minimally Invasive Surgery (MIS) in Paediatric Mediastinal Masses and Thoracic Tumours. MIS has transformed paediatric surgical oncology. This chapter explores the pivotal role of MIS in managing thoracic and mediastinal tumours in children, emphasising diagnostic and therapeutic advancements. Video-assisted thoracoscopic surgery has demonstrated significant utility, allowing for precise tumour resections and reduced morbidity. The techniques' feasibility and efficacy are underscored across a range of tumour types, including thymic, neurogenic and germ cell tumours, with promising outcomes in both high-resource and low- and middle-income countries (LMICs). The chapter pretends to be a practical guide for surgeons treating children with mediastinal and thoracic tumours, describing types of neoplasms, diagnostic approaches and treatment principles and options, with particular focus on surgical nuances and intraoperative advice. Despite its benefits, the chapter highlights critical challenges, including the limitations posed by large, invasive tumours and resource constraints in LMICs. The role of MIS in pulmonary metastases, particularly in osteosarcoma, is also discussed, with a focus on the balance between minimally invasive approaches and open surgeries for complete resection. Key principles for patient selection, surgical planning and the integration of advanced imaging and technology are emphasised, aiming for optimal outcomes. The chapter also addresses contraindications, different surgical techniques, anaesthetic considerations and the importance of global collaboration to expand access to MIS. It concludes with a call for continued innovation and equitable distribution of minimally invasive technologies worldwide, ensuring that the benefits of these techniques are accessible to all children with cancer, irrespective of geographical and economic barriers.

## Introduction

Minimally invasive surgery (MIS) has become an essential component in paediatric surgical oncology, offering several advantages over open surgery, such as significant reductions in hospital length of stay, complication rates, intraoperative blood loss and transfusion requirements, without compromising oncological outcomes [1]. Among MIS techniques, video-assisted thoracoscopic surgery (VATS) stands out for its availability, versatility and utility in managing thoracic and mediastinal tumours in children. Robotic-assisted thoracoscopic surgery (RATS) is also gaining popularity. Diagnostic and therapeutic indications have expanded alongside a better understanding of cancer biology, enhanced imaging for staging, 3D modeling for preoperative planning, refinement of MIS technologies, fluorescence-guided surgery and improved paediatric surgical oncology expertise [2]. However, challenges remain in global implementation, particularly in low- and middle-income countries (LMICs), where access to care is often limited and high-technology solutions may not be available [3].

## MIS indications in paediatric solid tumours

MIS plays both a diagnostic and therapeutic role in the management of paediatric thoracic and mediastinal tumours.

### Diagnostic biopsies

VATS provides safe and minimally invasive access to thoracic tumours for tissue sampling, ensuring accurate histopathological diagnosis while reducing surgical morbidity [2]. It allows for safe, direct-vision biopsy of lung parenchyma and pleura, chest wall, diaphragm and mediastinal lesions. Diagnostic challenges often arise in distinguishing between benign (infectious or inflammatory) lesions and metastatic disease in the lung parenchyma, pleural surfaces and mediastinum. This is especially relevant in high-risk paediatric cancer patients, such as those with solid tumours of unfavourable biology that often metastasise to these sites. The ability to obtain adequate tissue samples for histological and immunophenotypic analyses has improved diagnostic accuracy and reduced the need for repeat procedures [4].

Patients undergoing hematopoietic stem cell transplantation are at significant risk of developing opportunistic infections, including fungal and parasitic lung infections, which may mimic malignant lesions on imaging [5]. Additionally, immunocompromised children receiving chemotherapy may develop atypical infections (tuberculosis, pneumocystis pneumonia or invasive aspergillosis), presenting as pulmonary nodules [5]. Prior lung infections can lead to granulomas or other structural changes that complicate imaging interpretation. These patients benefit greatly from VATS for diagnosis due to their poor healing capacity.

In patients with suspected or confirmed metastatic disease, MIS may also be used for staging and assessment of disease progression.

### Therapeutic resections

VATS is indicated for the resection of most localised tumours of the mediastinum, both anterior (e.g., germ cell tumours and thymomas) and posterior (e.g., neurogenic tumours), as well as selected primary early-stage tumours without significant invasion into adjacent structures. In select patients, MIS can be feasible for metastatic lung lesions as well [6]. For chest wall tumours, thoracoscopy can aid in tumour resection, guide incision planning and extent of resection.

## Feasibility and outcomes of MIS in mediastinal tumours in children

MIS, particularly VATS and RATS, has gained prominence in the management of mediastinal tumours in paediatric patients. These tumours, often located in anatomically complex regions, pose challenges for surgical access and safe resection. MIS has demonstrated advantages over traditional open approaches, including faster recovery, less pain and better cosmetic outcomes, with increasing evidence supporting its feasibility, safety and efficacy.

A recent meta-analysis, including 333 paediatric patients, found that VATS is successful in achieving complete tumour excision with low perioperative morbidity, as well as decreased length of stay, complication rate, intraoperative blood loss and transfusion requirements compared to open thoracotomy [2]. Complications occur in 2%–20% of cases, but are generally minor and amenable to non-operative management, such as transient pneumothorax or atelectasis [7]. For experienced surgeons, serious complications (vascular injury) occur in up to 10% of patients with neurogenic posterior mediastinal tumours and positive image-defined risk factors [8].

### Specific tumour types and outcomes

**Figure 1.**
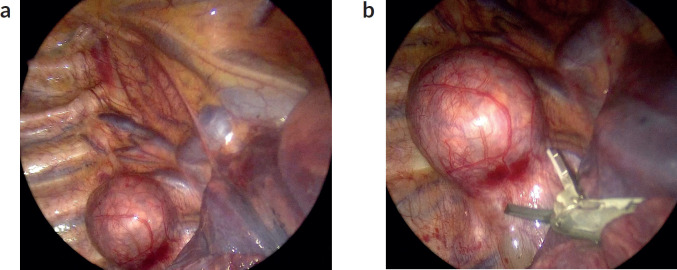
(a): Thoracoscopic view posterior mediastinal tumour. Thoracoscopic image depicting a neuroblastic posterior mediastinal tumour, at the right para-vertebral sulcus. A spherical lesion that extends over two vertebral bodies is evident. The lung is retracted medially (bottom right of the screen). The azygous vein can be seen cephalad and medial to the tumour, draining in the superior vena cava. Ribs and intercostal bundles are shown lateral of the lesion (left of the screen). (b): The lesion is resected with the use of an energy device (harmonic scalpel). VATS magnification aids in meticulous dissection.

1. Thymic tumours: MIS is increasingly used for thymectomies in paediatric patients, especially in early-stage thymomas or thymic hyperplasia. Outcomes are excellent, with complete resection rates exceeding 95% in experienced centers [9].
2. Neurogenic tumours: Typically located at the posterior mediastinum, these are especially amenable to MIS due to their peripheral location, solid consistency, limited vascularity and frequent lack of involvement with vital structures (Figure 1a and b), especially in older children, since they tend to be well-differentiated. Of course, a large posterior mediastinal neuroblastoma encasing the aorta or with thoraco-abdominal extent is likely not a suitable candidate for an MIS approach. However, in well-selected cases, studies report VATS with excellent outcomes, low recurrence rates and minimal perioperative morbidity [10].
3. Teratomas and germ cell tumours: Mature teratomas and benign germ cell tumours in the anterior mediastinum are amenable to MIS resection [1]. Malignant or extensive germ cell tumours, involving adjacent vital structures [11] require careful selection, as incomplete resection will affect prognosis [11, 12].
4. Lymphomas: In addition to interventional radiology-guided core biopsies, VATS has become a useful technique for obtaining tissue samples in patients with suspected mediastinal lymphomas. The approach provides high diagnostic accuracy as more tissue can be obtained with minimal surgical trauma, facilitating timely initiation of systemic therapy [1, 13].

## Feasibility and outcomes of MIS in paediatric patients with lung metastases

Approximately 25% of children and adolescents with extracranial solid tumours present with metastases at diagnosis, and an additional 20% will develop metachronous pulmonary lesions during or after therapy [14]. The lungs are the most common site of metastatic spread across most paediatric solid tumours. While systemic therapy remains the cornerstone of treatment, long-term survival varies widely (20% to 70%) depending on tumour histology, biology and responsiveness to treatment [14]. Despite significant improvements in localised disease outcomes, prognosis remains poor for patients with metastatic tumours, particularly when lesions are refractory to chemotherapy or radiotherapy.

Surgical resection of pulmonary metastases serves both diagnostic and therapeutic purposes. It is most beneficial in tumours resistant to systemic therapies, where complete metastasectomy may offer a meaningful survival benefit. Conversely, in patients with disseminated, uncontrolled extra-pulmonary disease, the role of lung metastasectomy becomes less clear [15]. The decision to perform pulmonary metastasectomy depends on the tumour’s biology and its responsiveness to chemotherapy, radiation or targeted therapies.

### Surgical objectives for pulmonary metastasectomy

Surgical planning should distinguish between diagnostic and therapeutic intent. Diagnostic purposes aim to obtain one or two representative nodules using the least invasive approach. For therapeutic resections, the goal should be to achieve an R0 resection through localised, non-anatomic excisions (e.g., wedge resection or enucleation with a small margin of healthy tissue), aiming to maximise preservation of lung parenchyma. Segmentectomies, lobectomies or pneumonectomies are feasible through MIS, but are rarely necessary and reserved for specific circumstances (e.g., large, central lesions adjacent to the hilum, in patients with systemic disease control and no other metastatic sites) (Table 1).

**Figure 2.**
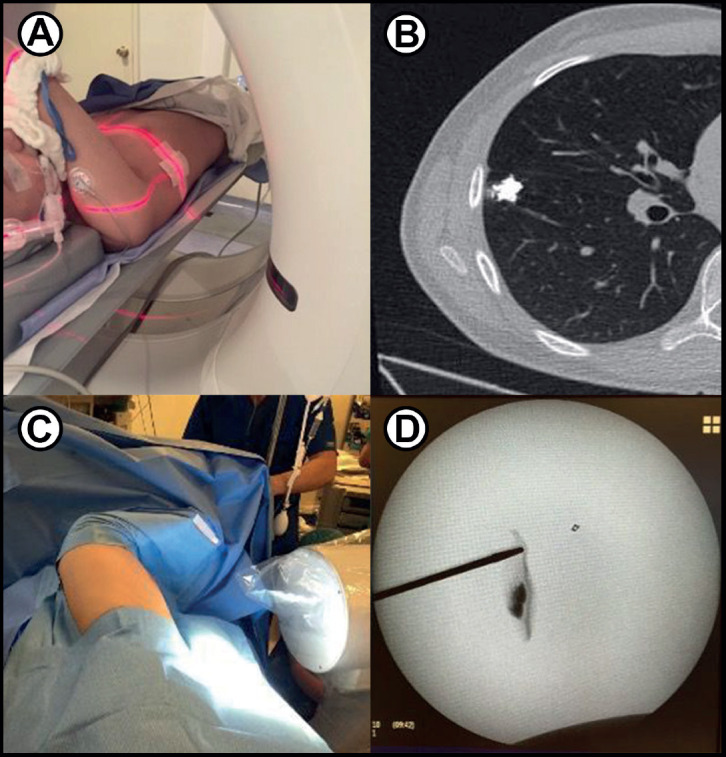
(a): Preoperative marking. A paediatric patient with osteosarcoma and a small right-sided lung nodule is marked under CT guidance. Just before surgery, interventional radiology injects 0.1 mL of lipiodol into the nodule to allow fluoroscopic visualisation during thoracoscopy. (b): Post marking CT image of the patient demonstrates the contrast-enhanced nodule. (c): During surgery, fluoroscopy aids in localising the lung nodule for full thoracoscopic resection. (d): Intraoperative fluoroscopy image identifies the small, intra-parenchymal nodule. (a–d) are courtesy of Gloria Gonzalez, MD. Paediatric Transplant and Oncology Surgeon, Cleveland Clinic.

A wide range of techniques has been described to localise deeper lesions and preserve maximal lung parenchyma. These include pre-operative marking with wires, coils or dyes and the use of intraoperative fluoroscopy, ultrasound or near infrared light [14] (Figure 2a–d).

Small, solitary, peripheral lung nodules (<1–2 cm), easy to visualise and resect, are particularly well-suited for thoracoscopic resection [16]. The ability to achieve negative surgical margins is a critical factor in determining the suitability of MIS. For solitary, peripheral lesions, recurrence rates and overall survival following MIS metastasectomy are similar to those achieved with open surgery, provided that negative margins are obtained [17].

For patients with bilateral disease, staged or simultaneous bilateral VATS with alternating one-lung ventilation can reduce the morbidity of formal thoracotomies [18] and allow for faster resumption of systemic chemotherapy. This approach is feasible when lesions are accessible via VATS and clear margins can be achieved. For patients with recurrent pulmonary metastases, VATS may offer a less invasive option for repeat resections, reducing postoperative pain and preserving pulmonary function [17].

## Particularities of MIS in metastatic osteosarcoma

Osteosarcoma is the most common solid tumour in children for which thoracic metastasectomy is considered. The role of thoracoscopic resection of lung metastases in patients with osteosarcoma is controversial. Complete resection of these nodules is critical for long-term survival. However, computed tomography (CT) scanning consistently underestimates the number of lung nodules in these patients and thoracoscopy, by the loss of tactile feedback, may not identify all lesions. One exception may be patients who completed chemotherapy and, during follow-up, present with solitary lung nodules on imaging. It has been shown that these particular patients are unlikely to have additional metastatic sites [19].

## Open versus thoracoscopic approach for pulmonary lesions in osteosarcoma

Patients diagnosed with osteosarcoma benefit from complete resection of all pulmonary metastases, as those achieving complete surgical remission may become long-term survivors [12]. To date, this is more feasible through open approaches, which allow for palpation and tactile feedback for detecting otherwise unidentifiable lesions by tomography.

Thoracoscopic and robot-assisted resections lack tactile feedback, making it challenging to intraoperatively locate small or intraparenchymal lesions. Currently, the role of minimally invasive techniques is limited to diagnostic biopsies of superficial nodules. The Children’s Oncology Group is conducting a protocol to validate the utility of thoracoscopic surgery in patients with metastatic osteosarcoma to the lung, limited to no more than four superficial lesions accessible via this approach [20] (Table 2).

Minimally invasive techniques may be considered for subsequent relapses of solitary pulmonary nodules diagnosed after completing therapy, as ipsilateral disease is unlikely to be found [19].

Patients diagnosed with osteosarcoma benefit from complete surgical resection of pulmonary metastases. This is more feasible through open approaches. However, additional studies on the utility of minimally invasive techniques are well justified. Surgery should be performed by an experienced surgical team, familiar with the surgical principles of osteosarcoma and must be effective, feasible and safe.

Non-surgical options such as stereotactic radiotherapy, radiofrequency ablation or cryotherapy may be considered as alternative options for patients who are not surgical candidates [21, 22].

## MIS contraindications

Tumour characteristics: Large or invasive tumours encasing vital structures such as the great vessels, esophagus or trachea, represent a relative contraindication for MIS, depending on the surgeon’s expertise. These may not be suitable for MIS due to technical difficulties in achieving a safe and complete resection [23]. Complex mediastinal tumours may take longer to excise during a minimally invasive approach when compared to open surgery, especially in centers with limited experience. The steep learning curve associated with MIS in mediastinal tumours underscores the need for specialised training and high-volume centers to optimise outcomes.

Regarding centrally located pulmonary metastases near critical structures, such as the pulmonary hilum or major vessels, are not ideal candidates for MIS due to the risk of incomplete resection or significant complications. A high level of expertise is required to take on this scenario safely.

Severe adhesions: Dense pleural or mediastinal adhesions from prior surgeries or infections can hinder the safe use of VATS [24].

Patient-specific factors: Severe cardiopulmonary compromise, which may preclude the use of single-lung ventilation, is a relative contraindication for thoracoscopic surgery [25]. Single-lung ventilation may be difficult to achieve in patients too small for a double-lumen endotracheal tube, although main-stem bronchus intubation with a single-lumen tube may suffice in these patients.

Individualised evaluation using imaging and multidisciplinary input is crucial for determining suitability for MIS.

## Surgical approach

### Types of minimally invasive approaches to the thorax

#### Thoracoscopy

**Figure 3.**
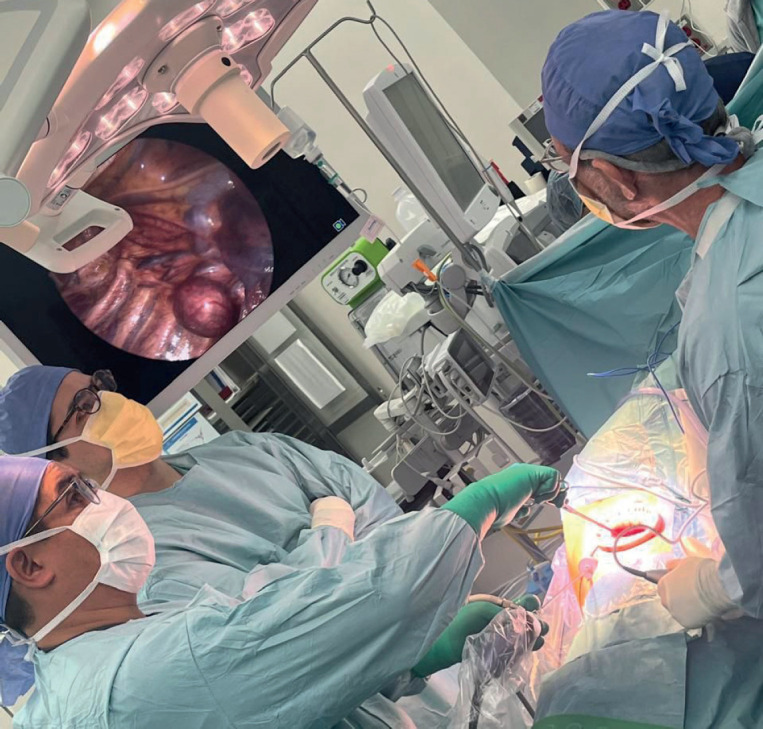
Surgical team setting. Surgical team positioning for effective minimally invasive access to the thorax and mediastinum

One view port and two working ports are typical. Best for ≤4 superficial lesions. Preoperative lesion marking is useful for this approach since there is no tactile feedback. Specimens are extracted in a bag [16]. It is generally used for diagnosis, obtaining pleural fluid or performing incisional pleural or lung biopsies (Figure 3).

#### Video-assisted thoracoscopic surgery

**Figure 4.**
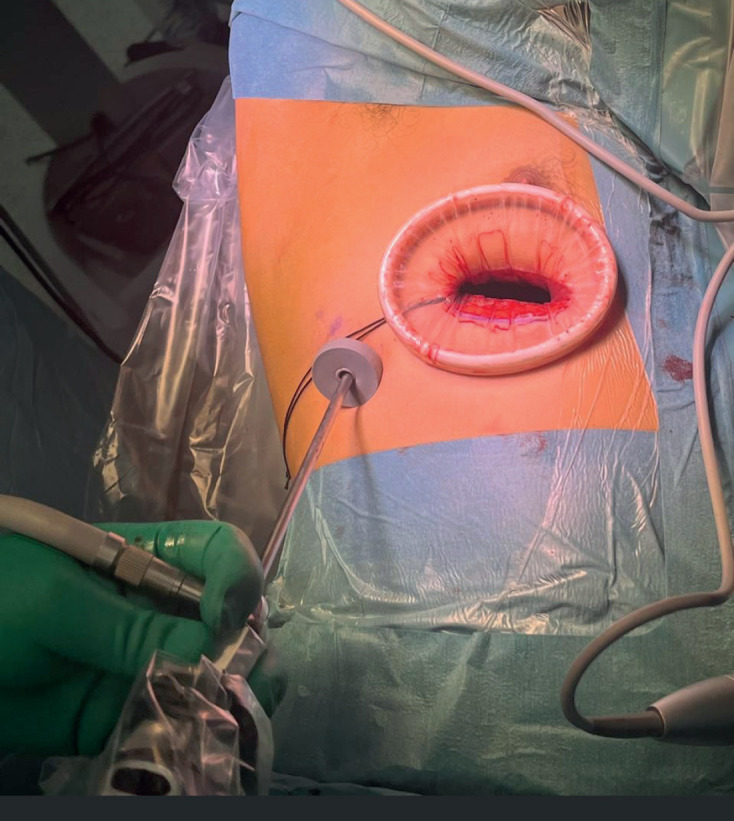
VATS access setting. Operative view of access in VATS. A camera is placed through a thoraco-port on the 7th intercostal space, at the mid-axillary line. The mini-thoracotomy extends 3–4 cm long from the anterior axillary line towards the sternum, usually at the fourth, fifth or sixth intercostal space, depending on the anatomic location of the mass. An Alexis wound retractor lubricated with sterile gel is placed into the mini thoracotomy in order to facilitate retraction of the lung using smooth instrumentation, and specimen extraction in a retrieval bag.

A 30-degree 5 mm camera is placed through a thoraco-port at the 7th intercostal space, at the mid-axillary line. After visual inspection of the pleural cavity and detailed identification of the lesion to be resected, a small incision will allow for effective instrumentation. The mini-thoracotomy extends 3–4 cm long from the anterior axillary line towards the sternum, at the inferior crease of the pectoralis major muscle. The thorax is accessed at the 4th or 5th intercostal space, depending on the particular tumour location. Incisions can be extended anteriorly or posteriorly if needed for vascular control, tactile feedback or *en-block* specimen extraction. A small Alexis wound retractor lubricated with sterile gel facilitates instrument retraction of the lung, smooth instrumentation and extraction of the specimen in a retrieval bag (Figure 4). Pulmonary lesions can be resected using a stapler (Endo-GIA). Liberation of the inferior pulmonary ligament allows for lung mobilisation and extra-thoracic resection of lower, peripheral lesions [18].

#### Robotic-assisted thoracoscopic surgery

Similar to VATS but utilising a robotic system. It allows for enhanced dexterity and visualisation in confined spaces. However, the cost is high and its availability is very limited in LMICs. For surgeons comfortable performing VATS, robotics facilitates the procedure. For those without previous VATS experience, there may be a significant learning curve [26].

### Anaesthetic considerations

Anaesthesia management is a critical component of MIS in paediatric patients with thoracic tumours and mediastinal masses. The unique challenges posed by these tumours, combined with the physiological impacts of MIS techniques, require careful planning and expertise.

**Figure 5.**
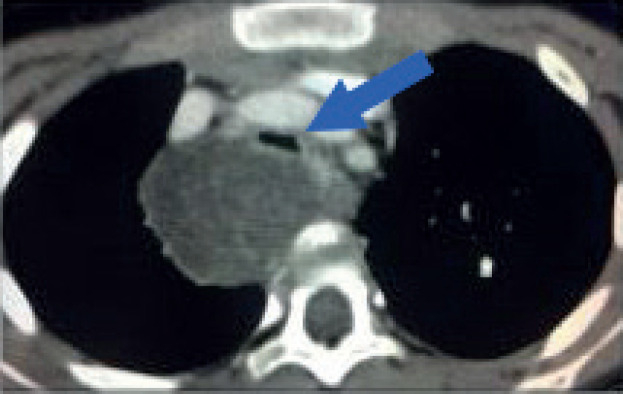
Tracheal compression. Contrast-enhanced axial CT image of a 6-year-old girl with a large tumour at the upper right posterior mediastinum. The lesion crosses the midline over the anterior surface of the vertebral body, at the level of the manubrium. Both, arterial and venous mediastinal great vessels are selectively enhanced with contrast. The trachea is severely compressed by the tumour (Blue arrow). This patient is at high risk of ventilatory collapse during the induction of anaesthesia.

Airway compression and cardiopulmonary function must be assessed preoperatively. Patients with clinical or radiological evidence of airway compression require pulmonary function tests (VEF1). Both pulmonary and cardiac function play a separate role in anaesthetic risk. For patients with airway compression and risk of ventilatory collapse during induction anaesthesia, it is critical to maintain spontaneous ventilation and avoid the use of muscle relaxants until the airway is secured (Figure 5). Flexible bronchoscopy may be a useful adjunct for difficult airway management or to confirm airway patency. On the other end of the spectrum, patients at high risk of cardiovascular collapse may require extracorporeal membrane oxygenation to tolerate the procedure.

### Patient positioning

Patient positioning is dictated by tumour location. Rolls paced under the thorax for extension are useful and a mild head elevation benefits the cardiopulmonary function.

- Lateral decubitus: commonly used for unilateral thoracic tumours.
- Supine positioning: preferred for anterior mediastinal mass resection because it provides optimal surgical access, facilitates airway management and allows for safe cardiopulmonary monitoring and support.
- 45º angled decubitus: useful for posterior mediastinal lesions.

### Incision and trocar placement

Trocar placement varies based on tumour location, patient size and body habitus. It is important to consider that in thoracoscopy, the classic optimal triangulation used in abdominal MIS does not necessarily apply.

- For visualisation of the entire thoracic cavity, a 5 mm camera port placed at the 6th or 7th intercostal space, midaxillary line is convenient.
- Placement of the rest of the ports (or incisions) is determined by the initial thoracoscopic findings and preoperative images.
- For anterior mediastinal tumours, trocars are positioned at the subxiphoid area and the lateral chest wall to facilitate access and visualisation. The intercostal space for working ports is selected under direct visualisation, depending on the particular tumour anatomy.
- For localised posterior mediastinal tumours, VATS offers excellent visualisation and direct access. This approach uses a camera in the midaxillary port and a 4 cm antero-lateral thoracotomy at the pectoral crease, where access is feasible through the 4th or 5th intercostal space.

### Surgical technique

Key steps include:

- Ideally, single lung ventilation should be used as a collapsed lung aids in visualisation and tissue handling. However, dual lumen endotracheal tubes are not available in smaller sizes for younger patients. Alternatively, a bronchoscopy-guided selective intubation or bronchial blocker is effective.
- CO_2_ insufflation into the chest cavity to aid in lung compression can be used in fully thoracoscopic procedures. Close communication between the surgery and anaesthesia teams is essential.
- Using advanced instrumentation with surgical sealing energy devices and staplers are optimal to minimise blood loss.
- The oncologic principle of *en bloc* tumour resection for certain tumour histologies cannot be overstated, particularly in MIS.

### Tumour resection

Panoramic and close-up visualisation of the operative field to guide precise tumour dissection. As mentioned, an Alexis wound retractor on the anterolateral thoracotomy incision (slightly lubricated with sterile gel) allows for smoother instrumentation and direct views. The incision can be extended for critical structure control, tactile feedback and versatility if needed. Ensure adequate hemostasis during every step of the procedure.

Image and fluorescence-guided surgery may be used for helping to help identify inconspicuous lesions and ensure free margin resections. A hook wire can be placed preoperatively by interventional radiology under CT-guidance to mark intraparenchymal lesions but it tend to dislodge easily. Indocyanine green (ICG) and other fluorescent dyes are promising adjuncts currently under investigation. ICG can be injected IV (24–72 hours prior to the procedure, depending on histology) or directly intralesional, by interventional radiology or the surgeon immediately before the procedure. Tissue must be exposed to near infrared light for the ICG to fluoresce. Methylene blue is used intralesional, but in the author’s experience (JS), it tends to disperse through the tissues and avoid proper visualisation of nodules.

The specimen is extracted in a bag through a small thoracotomy incision and should be properly handled, marked and referenced.

A chest tube can be placed at the surgeons´ discretion. If used, it can be delivered through the camera port site.

Intercostal nerve blocks or radiofrequency rhizolysis reduce postoperative opioid consumption, rapid resumption of activity and shorten hospital length of stay.

### Tips and pitfalls

Tips:

- Careful patient selection and preoperative planning.
- Delicate tissue handling.
- Employing operative technology (proper visual capacity, energy devices and staplers to minimise blood loss) and visual aids (ICG, fluoroscopy or ultrasound) enhances tumour localisation.

Pitfalls:

- Know when to convert.
- Be prepared to recognise the distorted anatomy as the procedure advances. Inadequate exposure or excessive manipulation of tumours may lead to bleeding or tumour spillage. Bleeding can be very sudden and aggressive.
- Plan for prompt conversion to open thoracotomy and have the appropriate instruments available.

## Outcomes in LMICs

In LMICs, MIS for mediastinal and lung tumours remains underutilised due to limited access to advanced surgical tools and expertise. Additionally, patients tend to present with more advanced disease, often requiring open thoracotomy. However, as training programs and infrastructure improve, MIS is increasingly being adopted, showing promising outcomes even in resource-constrained settings. Recent studies highlight the importance of international collaboration to expand access to MIS for paediatric mediastinal tumours globally [26].

## MIS in global paediatric surgical oncology

MIS has the potential to bridge gaps in paediatric oncology outcomes worldwide. However, LMICs face several barriers:

- Limited access to advanced MIS equipment and trained personnel [3].
- High costs associated with procuring and maintaining MIS technologies.
- Challenges in perioperative care due to resource constraints.

Collaborative efforts, such as international training programs and the donation of MIS tools, are crucial to improving global equity in paediatric oncology care.

## Conclusion

The role of MIS in managing mediastinal and thoracic tumours is well-established in paediatric surgical oncology. Benefits include high resection rates (>90%), low complication rates and favourable oncological outcomes [8]. As technology evolves, indications continue to broaden. However, efforts are needed to ensure that these benefits are accessible globally, particularly in LMICs. Future research should focus on refining techniques, developing cost-effective solutions and evaluating long-term outcomes. Finally, careful patient selection is paramount, considering tumour size and histology, location, the patient's habitus and cardiopulmonary reserve.

## Conflicts of interest

All the authors declare that they have no conflicts of interest.

## Funding

All the authors declare no funding for this research.

## Figures and Tables

**Table 1.** 

Principles for pulmonary metastasectomy
Consider tumour histology and biological behaviour. Accurate diagnosis of pulmonary lesions may avoid unnecessary toxic therapies. The number of metastases or disease-free interval are not contraindications for metastasectomy. Bilateral synchronous or staged resections are well tolerated. Negative margins are essential; localised resections should be performed to preserve as much adequate lung volume as possible [16].

**Table 2.** 

Basic principles for osteosarcoma surgery.
a) Primary tumour control has been achieved (or is achievable). b) There is no clinical or radiological evidence of unresectable extra thoracic metastases. c) Postoperative pulmonary function is anticipated to be adequate. d) The patient’s clinical condition and functional status are acceptable. e) Clinical and radiological findings indicate that complete resection is feasible.
